# Editorial introduction: animal models relevant to mental health disorders

**DOI:** 10.1042/ETLS20220094

**Published:** 2022-12-09

**Authors:** Amy L. Milton

**Affiliations:** Department of Psychology, University of Cambridge, Cambridge, U.K.

**Keywords:** mental health, research domain criteria, translational science

## Abstract

Mental health disorders affect a substantial proportion of the worldwide population, and currently available treatments do not work for all affected individuals. Understanding the psychological and biological mechanisms that underlie mental health disorders will facilitate treatment development, and the use of translational animal models is potentially transformative for this. Structured around the US National Institute of Mental Health's ‘Research Domain Criteria’ (RDoC) approach, this special issue showcases reviews that consider how animal models can best be used to understand and treat the processes that go awry in mental health disorders.

Mental health disorders affect a substantial proportion of the global population [1], with estimates suggesting that in 2019, one in eight people worldwide were living with a mental health disorder. Mental health disorders produce profound costs on both society and the individual; not only in terms of economic costs [2,3] but also on quality of life. While there are some effective pharmacotherapies and cognitive and behavioural therapies available to treat mental health disorders, these are efficacious for just a subset of patients [4], underlining the importance of new treatment development.

Mental health disorders are clinically diagnosed by reference to the Diagnostic and Statistical Manual of Mental Health Disorders (currently on its fifth edition, DSM-5; [5]), which provides a list of symptoms that need to be experienced by an individual in order to be diagnosed with a specific type of mental health disorder. In 2010, the US National Institutes of Mental Health put forward a new framework for understanding mental health disorders, the Research Domain Criteria (RDoC; [6]). The aim of the RDoC approach was to move away from understanding mental health disorders as clusters of symptoms, instead focusing on underlying psychological and biological constructs that, in specific combinations, give rise to mental health disorders. The RDoC framework emphasises understanding these specific domains at different levels of analysis (from genes to behaviour) with the view that behaviour is on a spectrum from functional to dysfunctional, rather than being dichotomous (satisfying the criteria for diagnosis, or not), consistent with the view of ‘dimensional psychiatry’ [7,8].

A major advantage of the RDoC and dimensional psychiatry approach is that it allows the deconstruction of mental health disorders into specific psychological and biological processes, which can be isolated for study. It also makes the study of mental health disorders more tractable in animal models, particularly when translational and backtranslational (or ‘reverse translational’) behavioural tasks can be used to study the same process in humans and animals [9,10]. The use of animal models relevant to mental health disorders has numerous advantages, including the capacity to conduct causal manipulations that would unfeasible and/or unethical in humans (e.g. brain-based interventions, or developmental manipulations that would increase the risk of developing a mental health disorder). Another advantage of the RDoC approach is that it allows for better understanding of *functional* behaviour and potentially of resilience, in addition to susceptibility, factors. The aim of this special issue is to provide an up-to-date, reference volume of articles showcasing animal models relevant to mental health disorders, with a particular focus on the RDoC domains of ‘negative valence systems’, ‘positive valence systems’ and ‘cognitive systems’.

It begins with a detailed analysis of the construct of fear by Monfils and Domjan [11], considering the transition between adaptive and maladaptive fear, and whether the ‘deceptively simple’ measures of fear usually taken in the laboratory might be enhanced by considering individual differences in fear learning. In the face of an uncertain threat, individuals tend more towards anxiety than fear; being sensitive to uncertain, potentially threatening stimuli within the environment. A key part of the neural circuitry underlying anxiety is the bed nucleus of the stria terminalis (BNST), which is the focus of the systematic literature review by Sherman et al. [12]. The BNST is key region considered also in the review by Fanselow [13], who critically examines the relationship between the fear, anxiety and ‘sustained threat’ RDoC constructs from the perspective of predator imminence theory. Arguing that the BNST may be recruited when threatening stimuli are more challenging to learn about, this review suggests that sustained threat may be more related to a shifting of the predator imminence continuum, rather than representing a separate construct.

Loss — of reward or other motivationally relevant stimuli, time-limited or sustained — is another key element of the RDoC ‘negative valence system’ domain. Loss of motivation for reward, or apathy, is the focus of the review by Jackson & Robinson [14]. Although apathy is a prominent symptom for many people living with major depressive disorder (MDD), Jackson and Robinson [14] consider how apathy is not specific to MDD, and how apathy and MDD can be distinguished at the clinical and neurocircuitry levels. They also discuss the importance of this distinction, particularly from the perspective of developing translational animal models that have relevance for MDD and other disorders in which apathy is a component symptom.

Loss of reward can be demotivating, but also frustrating. Frustrative non-reward, and its relationship to the development of compulsive behaviour, is the topic of the review by Moreno-Montoya et al. [15]. They consider the behaviour of schedule-induced polydipsia (SIP), in which individuals are exposed to intermittent small rewards, inducing a state of frustration and the development of coping behaviours. In subset of the population, these coping behaviours can become excessive and maladaptive, and possibly compulsive. Moreno-Montoya et al. [15] consider the psychological differences between high compulsive individuals and the general population, and whether these individuals vary in their processing of negatively valenced stimuli, or whether the behavioural changes reflect alterations in cognitive constructs.

The focus of the issue then turns to the ‘positive valence system’, with articles by Burton and Balleine [16] and Salamone and Correa [17] considering different aspects of reward learning and motivation. Burton & Balleine [16] consider the different systems supporting learning about rewards, and how these interact to produce functional, integrated and adaptive behaviour, while also being independent processes and sometimes directly competing with each other for dominance of behaviour. Salamone and Correa [17] consider the valuation of reward, and particularly the subconstruct of effort, or willingness to work, for reward. They argue that while the RDoC approach has strength in supporting the study of more complex behaviours that have greater translational relevance to effort-based choice measures in humans, it also has limitations. In particular, the categorisation effort-based choice as a subconstruct of ‘positive valence systems’ does not do justice to the interaction with the ‘negative valence systems’ that also have relevance to these decisions and are required for the emergence of integrated and adaptive behaviour.

The final section of the issue turns to ‘cognitive systems’. Roberts & Young [18] consider the importance of developing treatments to ameliorate attentional deficits across numerous mental health disorders, and how the development of translational rodent behavioural tasks can facilitate this. Reviewing historical and more recent tasks, they emphasise the advantage of these translational approaches, and also discuss the limitations of these highly cognitively demanding tasks, which often require extensive training. Requiring little training, and indeed capitalising on the spontaneous and automatic nature of learning, are tasks that attempt to model episodic memory in humans, as reviewed by Castelo-Branco and Barbosa [19]. Noting that the study of episodic memory is challenging in non-verbal animals, they discuss the development of tasks that aim to capitalise on rodents’ natural exploratory behaviour, while also satisfying the combination of ‘what’, ‘where’ and ‘when’ that would satisfy a definition of ‘episodic-like memory’ in animals [20].

There is a clear need for better understanding of mental health disorders, and for new treatment development. Focusing on the psychological and neurobiological processes that go awry in mental health disorders may allow for better focusing of treatment onto ameliorating dysfunctional processes, regarding of the specific disorder that has been diagnosed for an individual. This understanding will be markedly facilitated by the development of highly translational animal models, and the wealth of papers within this special issue illustrate the quantity, and the quality, of research that aims to address this.

## Open Access

Open access for this article was enabled by the participation of University of Cambridge in an all-inclusive *Read & Publish* agreement with Portland Press and the Biochemical Society under a transformative agreement with JISC.

## Abbreviations

BNST: bed nucleus of the stria terminalis
MDD: major depressive disorder
RDoC: Research Domain Criteria
